# The effect of training and *Tribulus terrestris* extract on the antioxidant system and telomere functional markers in the liver tissue of rats exposed to stanozolol

**DOI:** 10.22038/ajp.2025.26403

**Published:** 2026

**Authors:** Najmeh Kiani, Saeed Keshavarz, Seyed ali Hosseini, Jamshid Banai

**Affiliations:** 1 *Department of Sports Physiology, Najafabad Branch, Islamic Azad University,Najafabad, Iran*; 2 *Department of Sports Physiology, Marvdasht Branch, Islamic Azad University, Marvdasht, Iran*

**Keywords:** Exercise, Tribulus terrestris, Oxidative stress, Telomere Function, Liver, Stanozolol

## Abstract

**Objective:**

The present study aimed to assess the effect of training along with administration of *Tribulus terrestris* (T) on the antioxidant system and telomere functional markers in the liver tissue of rats exposed to stanozolol.

**Materials and Methods:**

Forty- nine male rats, with average age and weight of 8-10 weeks and 180-220 g respectively, were randomly divided into 7 groups of seven rats: 1) Sh: Sham, 2) S: stanozolol, 3) S+T50: stanozolol+ 50 mg/kg *T. terrestris*, 4) S+T100: stanozolol+ 100 mg/kg *T. terrestris*, 5) S+RT: stanozolol + resistance training, 6) S+RT+T50: stanozolol + resistance training + 50 mg/kg *T. terrestris*, and 7) S+RT+T100: stanozolol + resistance training + 100 mg/kg *T. terrestris*. Rats in the S groups received 5 mg/kg stanozolol intraperitoneally (25 mg/kg/wk). Groups 5 (R+T), 6 (S+RT+T50), and 7 (S+RT+T100) performed resistance trainings three sessions per week with an intensity of 30-100 percent of body weight for eight weeks. Also, groups 3 (S+T50), 4 (S+T100), 6 (S+RT+T50) and 7 (S+RT+T100) received daily ethanolic extract of T with doses of 50 and 100 mg/kg intraperitoneally.

**Results:**

In the S+RT, S+T50, S+T100, S+RT+T50, and S+RT+T100 groups, malondialdehyde (MDA) levels were significantly lower and gene expression of phosphoinositide 3-kinases *(PI3K*), protein kinase B (*Akt*), and telomerase reverse transcriptase (*TERT*) levels were higher than the S group. Also, *phosphatase and tensin homolog* (*PTEN*) and *TERT* gene expression levels in the S+T100 group were significantly higher than the S+T50 group.

**Conclusion:**

Training and T have a positive effect on the transcription pathway of antioxidants and telomere protection.

## Introduction

Anabolic androgenic steroids (AAS) are a group of endogenous androgens and testosterone derivatives that are widely used to increase muscle mass in athletes (Petrovic et al. 2022). Studies showed that AAS are used by both professional and amateur athletes as well as in non-athletes in order to increase physical performance, muscle mass and strength (Agha et al. 2022). Although AAS are also occasionally used for therapeutic purposes, their abuse has resulted in various damages such as metabolic, cardiovascular, nervous system, kidney, and liver disorders (Agahi and Salehi 2022; Agha et al. 2022; Petrovic et al. 2022). According to previous empirical findings, AAS abuse is likely to lead to an increase in the storage of lipids, disruption of liver enzymes, and free radicals produced by mitochondria. Therefore, increasing the damages to lipids, structural proteins of the cell and the DNA of liver cells as well as reducing the antioxidants such as superoxide dismutase (SOD) and glutathione peroxidase (GPx) can be partially attributed to the abuse of these drugs. It also increases lipid peroxidation marker malondialdehyde (MDA) (Sadasivam et al. 2022). In addition, researchers believe that the abuse of AAS in animal samples disrupts biological pathways and induces oxidative stress, leading to damage to the chromosomal structure. In other words, free radicals lead to disruption of the expression of *phosphatase and tensin homolog (PTEN)* and *telomerase reverse transcriptase* (*TERT)* proteins and this happens through the pathway of disruption in the gene expression of *protein phosphatase 2 (PP2A)*, *protein kinase B (PKB/Akt)* as well as *phosphatidylinositol 3-kinase (PI3K)*, and finally, cell lifespan decreases with the shortening of the telomere bases in the chromosome (Kotil and Mehtap 2022; Seo et al. 2022). 

In line with the research trend, *in vitro* and *in vivo* studies show that increasing the ratio of oxidative stress to antioxidants by disrupting *PTEN* and *TERT* leads to the shortening of telomere length (Armstrong 2023). researchers showed that supra-physiological dose of stanozolol (S) in rats with and without swimming training led to disturbances in the renal *PI3K*/*AKT*/*PTEN* pathway (Kotil and Mehtap 2022) . Despite the abundance of research studies in the field of irreparable damage of AAS in various organs, these drugs are still used for specific purposes in athletes and non-athletes; therefore, researchers believe that the use of medicinal plants along with AAS can partially reduce their harm (Agahi and Salehi 2022). 

*Tribulus terrestris *(T) has been used in traditional medicine for a long time due to its antioxidant properties, and for improving sex hormones, improving lipid metabolism, and treating anemia and liver disorders (Al-Eisa et al. 2022). It is believed that this plant, due to its saponins, alkaloids, flavonoids, natural oils and resins, can neutralize free radicals and strengthen the antioxidant system (Al-Eisa et al. 2022). Studies have shown that consumption of T leads to an increase in SOD, GPx, catalase, glutathione S transferase (GST) as well as decreases in interleukin-1 beta (IL-1β) and tumor necrosis factor alpha (TNF-α) *in vitro* and in liver tissue of diabetic rats (Al-Eisa et al. 2022; Khalid et al. 2022) . Also, in a study, researchers showed that T extract along with exercise significantly decreased MDA and cytochrome C, and increased *O-6-methylguanine*-DNA methyltransferase (MGMT) in kidney and heart tissues following exposure to arsenic and hydrogen peroxide (Delfani and Matin Homaee 2021; Farokhi et al. 2022) . Despite the protective effects of T consumption in various tissues against oxidative stress and DNA damage, the protective mechanism of this medicinal plant against telomere damage and the *Akt/PI3K/PTEN* pathway, especially in liver tissue, is still not well known. On the other hand, considering the increase in the abuse of anabolic steroids and the resulting liver damage, it seems necessary to conduct fundamental studies in this field to better understand the changes in oxidative stress and telomere damage. 

Therefore, the present study aimed to investigate the effect of eight weeks of resistance training along with T administration on the antioxidant system and telomere activity in the liver tissue of rats exposed to S.

## Materials and Methods

### Animal housing

In this experimental study, 49 Sprague-Dawley rats with an approximate weight of 200 g and age of 8-10 weeks were purchased from the Laboratory Animal Breeding and Reproduction Center of Islamic Azad University, Marvdasht branch. Then rats were kept in the laboratory environment for seven days for adaptation to the new environment. It is worth mentioning that during the entire research period, the ethical principles of working with animals were maintained according to the Helsinki Treaty and under the supervision of the Institutional Review Board of Islamic Azad University, Najafabad Branch, with the approved code IR.IAU.NAJAFABAD.REC.14010180. In addition, the animals were kept under standard conditions in terms of temperature (22-24°C), light (12-hr light and dark cycle), and relative humidity of 55%, in polycarbonate cages with autoclave capability. After the adaptation period, on the 8th day, the rats were randomly divided into 7 groups and 7 rats in each group including: (1) Sham group (Sh), that received normal saline, (2) Stanozolol group (S), (3) Stanozolol + 50 mg/kg *T. terrestris* (S+T50), (4) Stanozolol + 100 mg/kg *T. terrestris* (S+T100), (5) Stanozolol + resistance training (S+RT), (6) Stanozolol + 50 mg/kg *T. terrestris* + resistance training (S+RT+T50), (7) Stanozolol + 100 mg/kg *T. terrestris* + resistance training (S+RT+T100).

### Stanozolol preparation

In this study, S was purchased from WEBER Company (USA). Then, rats in groups S, S+T50, S+T100, S+RT, S+RT+T50 and S+RT+T100 received S 5 mg/kg intraperitoneally. It is worth noting that in this research, the rats received a cumulative dose of 25 mg/kg on 5 consecutive days (Dos Santos et al. 2013; Agahi and Salehi 2022).

### Tribulus terrestris preparation

*Tribulus terrestris* (a native of Fars province, Iran) was prepared from Jihad of Agriculture (Fars province, Iran) with herbarium code 3224. To prepare T extract, first the fruit of this plant was ground, then 100 g of the powder was placed in 80 ml of 70% ethanol, and then this solution was kept in the laboratory for 3 days. After three days, the solution was first filtered through paper, then the liquid was separated using a vacuum purification device, and the dried extract of *Tribulus terrestris *was obtained. Further, after concentrating the extract with normal saline, the rats received doses of 50 and 100 mg/kg intraperitoneally (Derakhshandeh et al. 2022)

### Resistance training protocol

Rats performed resistance trainings using a ladder with a height of one meter, the distance between the steps was 4 cm and the slope was 85 degrees, so that the resistance trainings started from 30% of body weight in the first week and increased to 100% of body weight. It is worth mentioning that in order to warm up at the beginning of the training sessions, the rats climbed the ladder four times without weights. Also, the trainings in each session included four sets (the first set was 50%, the second set was 75%, the third set was 90% and the fourth set was 100% of the weight set for that week) and two repetitions (climbing the stairs twice). The interval between each set was 2- 3 min and the interval between each repetition was 40- 60 seconds (Derakhshandeh et al. 2022)

### Sampling

Forty-eight hours after the last training and supplementation session, the rats were fasted for 12-hr prior to the procedure..anesthesia was induced via intraperitoneal injection of ketamine (55 mg/kg) and xylazine (25 mg/kg) manufactured by Alfasan Company (Netherlands). To ensure complete anesthesia and lack of pain, rats were tested with tail and foot touch test for pain reflexes. Then, the abdominal cavity of the rats was carefully split and the liver tissue was carefully extracted by laboratory experts. After weighing and washing, the liver tissue was immediately immersed in liquid nitrogen and kept at -70 for transfer to the laboratory.

### GPx and MDA measurement in liver tissue

To measure the variables, first the liver tissue was pounded in a special oven, and after ensuring homogenization of the tissue and removal of damaged cells, GPx was measured using the Navand kit protocol, Version 0.8.1, on a scale of mU/ml. Also, MDA was measured by ZellBIO kit (Germany) CAT No. ZB-MDA-96A with a sensitivity of 0.1 μM.

### Akt, PI3K, PTEN and TERT measurement in liver tissue

qReal Time PCR method was used to measure the gene expression levels of *Akt, PI3K, PTEN *and* TERT *in liver tissue. For this purpose, first, 50 mg of tissue was separated from the liver, and RNA extraction was performed from the tissues, according to the manufacturer's protocol (Qiagen, Germany). To ensure the quality of RNA, electrophoresis was performed using agarose gel and using optical absorption property at 260 nm wavelength with a Sigma PicoDrop device (America). In addition, the formula (C (μg/μl) = A260 × ε × d/1000) was used to check the quality of RNA. Next, after the synthesis of cDNA using the manufacturer's protocol in the fermentase kit (K1621) and using the designed primers (Table 1) based on the guide of research genes on PubMed, the reverse transcription reaction was performed. It is worth mentioning that Allele IDv7.8 software was used to design the primers. In order to determine the efficiency and specificity of the primers, the pre-primers were evaluated using the software available on the NCBI website, and to measure the gene expression levels of the research variables, the internal control gene B2m was used and after ensuring the completion of the work of qReal Time PCR machine and after the samples reached the expression threshold (Cycle Threshold); to quantify the ratio of the desired gene to the reference gene, 2^-ΔΔCT^ formula was used. 

**Table 1:** Primers used in the study

	**Primer Sequences**	**Sizes (bp)**
*PI3K*	Forward: 5’-AGAGTTTCCTGGGCATCAATAA -3’	127
	Reverse: 5’-CTAACGCAGACATCCTGGAAT-3’	
*Akt1*	Forward: 5’-TCGTGTGGCAAGATGTGTAT-3’	102
	Reverse: 5’-GAGCTGTGAACTCCTCATCAA-3’	
*TERT*	Forward: 5’-TGTGATTCGGCTTCCCTTTG -3’	128
	Reverse: 5’-CCCTTAGTGACACTCCTGGAT-3’	
*PTEN*	Forward: 5’-GGAAAGGACGGACTGGTGTAA-3’	199
	Reverse: 5’-AGTGCCACTGGTCTGTAATCC-3’	
*B2m*	Forward: 5’-CGTGCTTGCCATTCAGAAA -3’	244
	Reverse: 5’-ATATACATCGGTCTCGGTGG -3’	

### Data analysis

In the descriptive statistics section, mean and standard deviation were considered. Shapiro-Wilk test was used to check the normality of data distribution. Considering the normality of data distribution, one-way analysis of variance was used to check the difference between groups. If the difference between the groups was significant, Tukey's *post- hoc* test was used to check and identify the location of the differences between the groups. The mean and standard deviation of GPx, MDA*, PI3K*, *Akt*, *PTEN* and *TERT *levels were analyzed in SPSS Graph Pad PRISM 8.3.3 software (p≤0.05). 

## Results

### Oxidant-antioxidants pathways analysis

**Figure 1:**
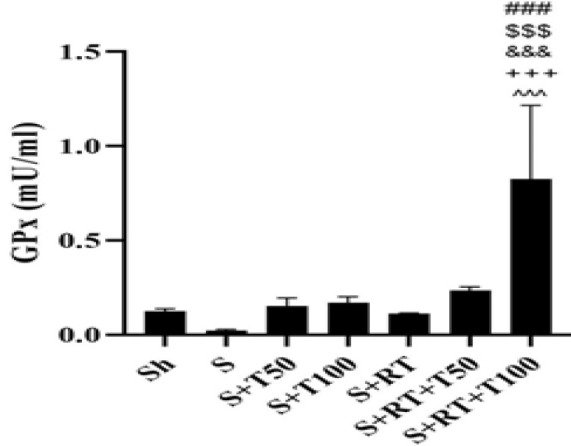
Glutathione peroxidases (GPx) levels in the liver tissue of rats in research groups (Sh: Sham, S: stanozolol, S+T50: stanozolol+ 50 mg/kg *Tribulus terrestris* , S+T100: stanozolol+ 100 mg/kg *Tribulus terrestris,* RT: resistance training, S+RT+T50: stanozolol+ resistance training + 50 mg/kg *Tribulus terrestris*, S+RT+T100: stanozolol+ resistance training + 100 mg/kg *Tribulus terrestris*). ###(p≤0.001) significant increase compared to the S group. $$$(p≤0.001) significant increase compared to the S+T50 group. &&&(p≤0.001) significant increase compared to the S+T100 group.+++(p≤0.001) significant increase compared to the S+RT group. ^^^(p≤0.001) significant increase compared to the S+RT+T50 group.

**Figure 2:**
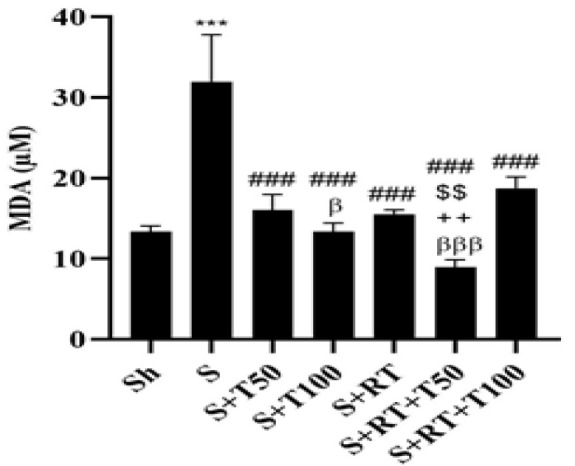
Malondialdehyde (MDA) levels in the liver tissue of rats in research groups (Sh: Sham, S: stanozolol, S+T50: stanozolol+ 50 mg/kg *Tribulus terrestris*, S+T100: stanozolol+ 100mg *Tribulus terrestris*, RT: resistance training, S+RT+T50: stanozolol+ resistance training + 50mg *Tribulus terrestris*, S+RT+T100: stanozolol+ resistance training + 100mg *Tribulus terrestris*). ***(p≤0.001) significant increase compared to Sh group. ### (p≤0.001) significant decrease compared to the S group. $$(p≤0.01) significant decrease compared to the S+T50 group. +++(p≤0.001) significant decrease compared to the S+RT group. βββ(p≤0.001) and β(p≤0.05) significant decrease compared to the S+RT+T100 group.

The results of the one-way analysis of variance (ANOVA) test revealed significant differences in GPx, MDA, *PI3K*, *Akt*, *PTEN,* and *TERT* levels among the research groups (p = 0.001). Tukey's post-hoc test indicated no significant difference in GPx levels between the Sh and S groups (p = 0.92). However, GPx levels in the S+RT+T100 group were significantly higher than those in the S, S+T50, S+T100, S+RT, and S+RT+T50 groups (p = 0.001) (Figure 1). MDA levels in the S group were significantly higher than Sh group (p=0.001); however, in the S+T50, S+T100, S+RT, S+RT+T50 and S+RT+T100 groups, MDA levels were significantly lower than the S group (p=0.001). MDA level was significantly lower in the S+RT+T50 group than the S+T50 group (p=0.0017). It was also significantly lower in the S+T100 group compared to the S+RT+T100 group (p = 0.02). Additionally, levels in the S+RT+T50 group were significantly lower than those in the S+RT (p = 0.003) and S+RT+T100 (p = 0.001) groups (Figure 2).

**Figure 3:**
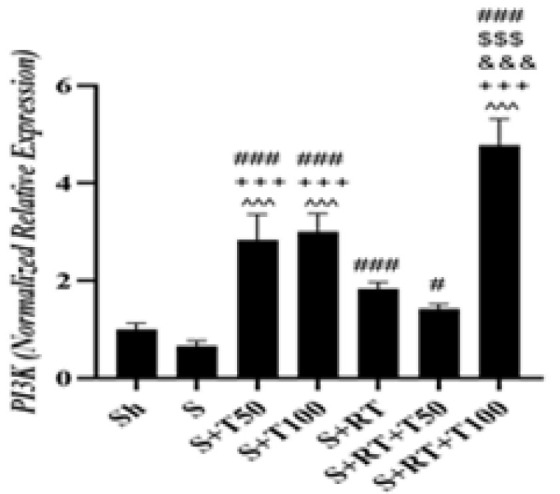
*Phosphoinositide 3-kinases(PI3K)* gene expression levels in the liver tissue of rats in research groups (Sh: Sham, S: stanozolol, S+T50: stanozolol+ 50 mg/kg *Tribulus terrestris* , S+T100: stanozolol+ 100 mg/kg *Tribulus terrestris*, RT: resistance training, S+RT+T50: stanozolol+ resistance training + 50 mg *Tribulus terrestris*, S+RT+T100: stanozolol+ resistance training + 100 mg/kg *Tribulus terrestris*).#(p≤0.05) and ### (P≤0.001) significant increase compared to the S group.$$$(p≤0.001) significant increase compared to the S+T50 group. &&&(p≤0.001) significant increase compared to the S+T100 group. +++(p≤0.001) significant increase compared to the S+RT group. ^^^(p≤0.001) significant increase compared to the S+RT+T50 group.

No significant difference was observed in *PI3K *level between the S and Sh groups (p=0.67); but *PI3K *level in the S+T50 (p=0.001), S+T100 (p=0.001), S+RT (p=0.001), S+RT+T50 (p=0.016) and S+RT+T100 (p=0.001) groups was significantly higher than the S group. It was also significantly higher in the S+T50 group than the S+RT (p=0.001) and S+RT+T50 (p=0.001) groups. However, it was significantly higher in the S+RT+T100 group than the S+T50 group (p=0.001). *PI3K *level in the S+T100 group was significantly higher than the S+RT (p=0.001) and S+RT+T50 (p=0.001) groups. However, it was significantly higher in the S+RT+T100 group than the S+T100 group (p=0.001). It was also significantly higher in the S+RT+T100 group than the S+RT (p=0.001) and S+RT+T50 (p=0.001) group (Figure 3). 

**Figure 4:**
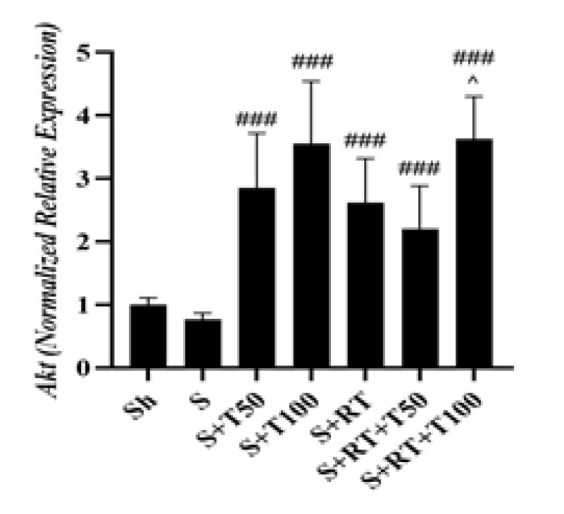
*Protein kinase B(Akt)* gene expression levels in the liver tissue of rats in research groups (Sh: Sham, S: stanozolol, S+T50: stanozolol+ 50 mg/kg *Tribulus terrestris* , S+T100: stanozolol+ 100 mg/kg *Tribulus terrestris*, RT: resistance training, S+RT+T50: stanozolol+ resistance training + 50 mg/kg *Tribulus terrestris*, S+RT+T100: stanozolol+ resistance training + 100 mg/kg *Tribulus terrestris*)###(p≤0.001) significant increase compared to the S group. ^(p≤0.05) significant increase compared to the S+RT+T50 group.

No significant difference was observed in *Akt* levels between Sh and S groups (p=0.99); but *Akt* levelin the S+T50 (p=0.001), S+T100 (p=0.001), S+RT (p=0.002), S+RT+T50 (p=0.03) and S+RT+T100 (p=0.001) groups was significantly higher than the S group. In addition, it was significantly higher in the S+RT+T100 group than the S+RT+T50 group (p=0.03) (Figure 4).

### Analysis of telomere activity

**Figure 5:**
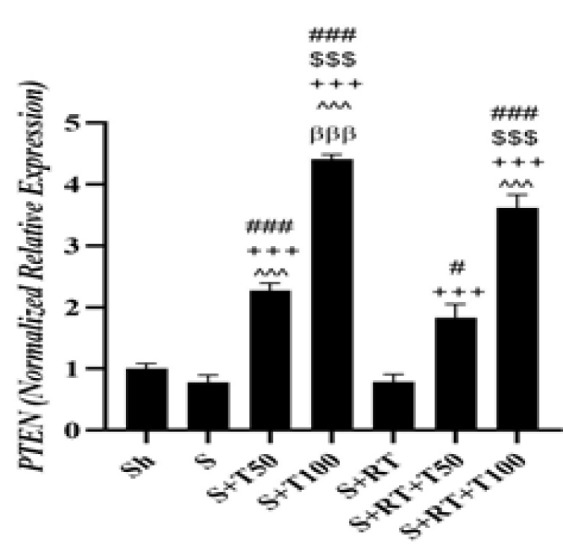
*Phosphatase and tensin homolog (PTEN)*gene expression levels in the liver tissue of rats in research groups (Sh: Sham, S: stanozolol, S+T50: stanozolol+ 50 mg/kg *Tribulus terrestris* , S+T100: stanozolol+ 100 mg/kg *Tribulus terrestris*, RT: resistance training, S+RT+T50: stanozolol+ resistance training + 50 mg *Tribulus terrestris*, S+RT+T100: stanozolol+ resistance training + 100 mg/kg *Tribulus terrestris*). #(p≤0.05) and ### (p≤0.001) significant increase compared to S group. $$$(p≤0.001) significant increase compared to S+T50 group. +++(p≤0.001) significant increase compared to the S+RT group. ^^^(p≤0.001) significant increase compared to S+RT+T50 group. βββ(p≤0.001) and β (p≤0.05) significant increase compared to S+RT+T100 group

No significant difference was observed in *PTEN *level between the Sh and S groups (p=0.24); but in the S+T50 (p=0.001), S+T100 (p=0.001), S+RT+T50 (p=0.03) and S+RT+T100 (p=0.001) groups, *PTEN *level was significantly higher than the S group. In addition, in the S+T100 (p=0.001) and S+RT+T100 (p=0.001) groups, *PTEN *level was significantly higher than the S+T50 group. But in the S+T50 group, it was significantly higher than the S+RT (p=0.001) and S+RT+T50 (p=0.001) groups. Also, *PTEN* levels in the S+T100 group was significantly higher than the S+RT (p=0.001), S+RT+T50 (p=0.001) and S+RT+T100 (p=0.001) groups. It was also significantly higher in the S+RT+T50 (p=0.001) and S+RT+T100 (p=0.001) groups than the S+RT group. In addition, it was significantly higher in the S+RT+T100 group than the S+RT+T50 group (p=0.001) (Figure 5).

**Figure 6:**
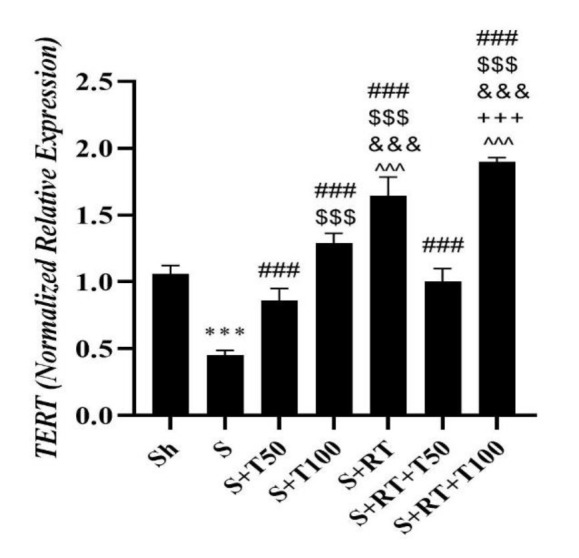
*Telomerase reverse transcriptase (TERT)*gene expression values in the liver tissue of rats in research groups (Sh: Sham, S: stanozolol, S+T50: stanozolol+ 50 mg/kg *Tribulus terrestris*, S+T100: stanozolol+ 100 mg/kg *Tribulus terrestris*, RT: resistance training, S+RT+T50: stanozolol+ resistance training + 50 mg *Tribulus terrestris*, S+RT+T100: stanozolol+ resistance training + 100 mg/kg *Tribulus terrestris*).***(p≤0.001) significant decrease compared to the Sh group. ### (P≤0.001) significant increase compared to the S group. $$$(p≤0.001) significant increase compared to the S+T50 group. &&&(p≤0.001) significant increase compared to the S+T100 group. +++(p≤0.001) significant increase compared to the S+RT group. ^^^(p≤0.001) significant increase compared to the S+RT+T50 group

*TERT *level in the S group was significantly lower than the Sh group (p=0.001); but in the S+T50 (p=0.001), S+T100 (p=0.001), S+RT (p=0.001), S+RT+T50 (p=0.001) and S+RT+T100 (p=0.001) groups, was significantly higher than the S group. It was also significantly higher in the S+100 (p=0.001), S+RT (p=0.001) and S+RT+T100 (p=0.001) groups than the S+T50 group as well as in S+RT (p=0.001), S+RT+T50 (p=0.001) and S+RT+T100 (p=0.001) groups was significantly higher than the S+T100 group. In addition, *TERT* levels in the S+RT group was significantly higher than the S+RT+T50 group (p=0.001); while in the S+RT+T100 group was significantly higher than the S+RT group (p=0.001). It was also significantly higher in the S+RT+T100 group than the S+RT+T50 group (p=0.001) (Figure 6). 

## Discussion

The findings of the current study depicted that the S group had, in comparison with Sh group, significantly higher MDA levels and significantly lower *TERT *levels. However, in the S+RT group, *MDA* levels were lower and *PI3K*, *Akt* and *TERT *levels were significantly higher than the S group. According to previous related findings, AAS abuse can lead to increased penetration of inflammatory cells into the liver, an increase in the number of Kupffer cells, and accordingly the enhanced activity of inflammatory cytokines (Petrovic et al. 2022). Moreover, it can cause collagen disruption and oxidative stress. . In particular, some scientists hold the belief that the disruption of mitochondrial function caused by AAS leads to an increase in free radicals in hepatocytes (through disrupting complexes I and III). Such malfunctioning, therefore, leads to increased damage to the cell structure such as DNA, phospholipids, pathological damage to the liver, disruption in liver enzymes, a decrease in antioxidant enzymes in the liver tissue such as SOD and GPx, as well as increased *MDA* levels (Petrovic et al. 2022). In addition, the increase of free radicals leads to the activation of the compensatory pathway to prevent DNA damage and the increase of *TERT* and *PTEN *via up-regulation of telomerase enzyme activity (Petrovic et al. 2022). In addition, the overregulation of this enzyme, regulated by the *PI3K/Akt* pathway, leads to an increase in *hTERT mRNA*, increase in telomerase activity, and disruption in *PTEN* function following steroid abuse (Kara et al. 2017). The number of studies on telomere damage following S abuse is, however, limited. For instance, Kara et al. who measured telomerase enzyme activity in peripheral blood mononuclear cells, found out that S consumption decreases telomere length and increases telomere enzyme activity and tail intensity (Kara et al.2017). They, however, concluded that exercise leads to modulation of telomerase enzyme activity. In our study, training led to the improvement of *TERT *activity functions as a regulatory protein in DNA synthesis using an RNA template. In another study by Ozcagli et al.*, *it was revealed that S abuse during exercise increases the expression of TERT in liver cells and has no significant effect on the expression of *PTEN (Ozcagli et al. 2018)*. The type of training in the study of (Kara et al. 2017; Ozcagli et al. 2018) was endurance training, which seems to have a more limited (not significant) effect on the *PI3K/Akt/mTOR* pathway than resistance training (Kara et al. 2017; Ozcagli et al. 2018). Having scant information about the signaling pathway following S exposure in liver tissue, selecting small sample sizes, using different measurement techniques, and utilizing different data analysis methods are possibly the main reasons for the contradiction in the results of the studies. The increase of GPx and the decrease of MDA following exercise were associated with the increase of *PI3K* and *Akt*, in this study, due the possible role of PI3K*/Akt* pathway in increasing the expression of antioxidants. In the S+T50 and S+T100 groups, MDA levels were lower and *PI3K*, *Akt*, *PTEN* and *TERT *levels were higher than the S group. Also, *PTEN* and *TERT *levels in the S+T100 group were significantly higher than S+T50. It is believed that T can improve antioxidant activity through regulating cellular redox due to the presence of saponins, quercetin, flavonoids and unsaturated fatty acids. In addition, the previous studies have revealed that the neutralization of O2 free radicals and hydrogen peroxide following the consumption of T can lead to an increase in the activity of deacetylases, sirtuins, the activity of cyclic adenosine monophosphate, *PI3K/Akt/NRF2* and finally increasing the expression of antioxidants. Therefore, this mechanism ultimately leads to an increase in GPx, Catalase, SOD and a decrease MDA in the liver tissue. Therefore, this mechanism ultimately leads to an increase in *GPx*, SOD and hence a decrease in MDA in the liver tissue (Al-Eisa et al. 2022). Furthermore, it appears that T through neutralizing free radicals leads to modulation of *O-6* *methylguanine* DNA *methyltransferase* (*MGMT*) (as a protective protein), reduction gene expression of *cytochrome C *(as an apoptotic marker), and reduction of *cyclooxygenase-2 *(*COX-2*) in liver and kidney tissues (Farokhi et al. 2021). Few studies examined the effect of T on telomerase, and *TERT* and *PTEN* activity; but in this context, the researchers showed that consumption of 10, 20, 40 and 80 mg/L T led to chromosomal protection in cells derived from human lymphocytes. Although the higher the dose was the more favorable they were, no significant difference was observed among different doses (Qari 2019). However, as supra- physiological consumption of T seems to have toxic effects on genetic and anti- estrogenic functions, researchers believe that supra- physiological doses can lead to transcriptional inhibition of antioxidants; Therefore, although T consumption has favorable effects, it should be used with caution and in conventional doses (Abudayyak et al. 2015). The doses used in the present study had same effect on *PI3K/Akt*, GPx and MDA related pathways. Although it is fairly apparent that genetic transcription such as *TERT* and *PTEN* in higher doses show a more genetic-protective effect, more research is needed in this field to have a deeper insight of the phenomenon. Similarly, the findings of Farokhi et al., and Delfani et al*.* showed that the consumption of T can increase DNA protection genes, reduce oxidative stress and inhibit apoptosis (Delfani and Matin Homaee 2021; Farokhi et al.2021). 

Derakhshandeh et al., and Arjmand et al. also stated that a higher dose of T has more positive effects on oxidative stress, lipid profile, and apoptosis in heart tissue of rats with S administration (Arjmand and Hosseini 2021; Derakhshandeh et al. 2022). 

In this study, it was also noticed that in the S+RT+T50 and S+RT+T100 groups, MDA levels were lower while *PI3K*, *Akt*, *PTEN* and *TERT* were significantly higher than the S group. We could not find a study that investigated the simultaneous effect of exercise and T on *TERT*, *PTEN* and telomerase activity in liver tissue. Therefore, one of the limitations of the current study is that its results cannot be safely compared to those of other studies. 

Nevertheless, in a similar context, Arjmand et al. reported that T consumption at the doses of 100 and 50 mg/kg with or without resistance training had anti-apoptotic effects in the heart tissue of rats exposed to S. In addition, the higher dose had a more favorable effect in such a way that the higher dose combined with resistance training had a synergistic effect on inhibiting apoptosis (Arjmand and Hosseini 2021). 

Another study also revealed that resistance training and T consumption with different doses improve lipid profile and apoptosis-related mechanisms the heart tissue of rats exposed to S (Derakhshandeh et al. 2020; Derakhshandeh et al. 2022) .

In general, it seems that resistance training with the mechanism of improving cellular redox and increasing *PI3K/Akt* expression leads to telomere protection (Kara et al. 2017; Ozcagli et al. 2018; Petrovic et al. 2022) and T protects cells and DNA via neutralization of O2 free radicals, hydrogen peroxide, activation of the *PI3K/Akt/NRF2* pathway, increase of transcription of antioxidants (Al-Eisa et al. 2022) and *MGMT*, as well as decrease of *cytochrome C* and *COX-2 *(Farokhi et al.2021) .

Considering the intended mechanisms, it seems that lack of measurement of telomerase activity is one of the limitations of the present study. Therefore, it is suggested that in future studies, telomerase enzyme activity, telomere length, tail intensity and liver tissue pathology be investigated to evaluate the function of hepatocytes. In addition, due to the contradictory findings of the correlative studies done on *TERT* and *PTEN*, the field turns out to be in a big need of more studies which consider the upstream and downstream pathways.

It appears that resistance training and *Tribulus terrestris* positively affect the transcription pathways of antioxidants and telomere protection. However, due to the limited existing research in this area, further studies are necessary to confirm these findings. 
